# Relationship between initial symptoms and the prognosis, sex, and demographic area of patients with COVID-19

**DOI:** 10.3389/fmed.2022.1040062

**Published:** 2022-12-14

**Authors:** Bárbara Oliván-Blázquez, Cruz Bartolomé-Moreno, Junkal Gericó-Aseguinolaza, Fátima Méndez-López, David Lerma-Irureta, Itziar Lamiquiz-Moneo, Selene Fernández-Martínez, Rosa Magallón-Botaya

**Affiliations:** ^1^Department of Psychology and Sociology, University of Zaragoza, Zaragoza, Spain; ^2^Group B21-20R, Health Research Institute of Aragon (IISA), Zaragoza, Spain; ^3^Network for Research on Chronicity, Primary Care and Health Promotion (RICAPPS) RD21/0016/0001, Zaragoza, Spain; ^4^Aragonese Healthcare Service, Zaragoza, Spain; ^5^Aragonese Healthcare Service, Department of Family and Community Care Teaching- Sector I, Zaragoza, Spain; ^6^Osakidetza-Basque Health Service, San Sebastian, Spain; ^7^Department of Medicine, Psychiatry and Dermatology, Faculty of Medicine, University of Zaragoza, Zaragoza, Spain; ^8^Miguel Servet University Hospital, Health Research Institute of Aragon (IISA), Zaragoza, Spain; ^9^Department of Human Anatomy and Histology, Faculty of Medicine, University of Zaragoza, Zaragoza, Spain; ^10^Group B17-20R, Health Research Institute of Aragon (IISA), Zaragoza, Spain

**Keywords:** signs and symptoms, sex, demographic area, COVID-19, prognosis, primary healthcare

## Abstract

**Background:**

A method of determining the initial symptoms and main prognostic identifiers for COVID-19 can be a key tool for physicians, especially primary care physicians. Therefore, the objective of this study was to examine the prognosis of patients with COVID-19 from two different demographic regions according to baseline and main symptoms, age, and sex.

**Methods:**

All individuals selected from both urban and rural health centers were over 18 years of age, had COVID-19 before 2 March 2021, and were followed up with a primary care physician. All patients included in this study were recruited in terms of sex, age at the time of infection, type of contact, baseline symptoms, primary and secondary symptomatology, emergency assistance, hospitalization, intensive care unit (ICU) admission, and death.

**Results:**

A total of 219 and 214 subjects were recruited from rural and urban health centers, respectively. Subjects with COVID-19 from rural areas were significantly older in age, with a higher proportion of men, and had significantly lower baseline and main symptoms than those from urban areas. In addition, the presence of both fever and dyspnea as the initial or main symptom is significantly associated with emergency assistance, hospitalization, and death, regardless of sex, age, and demographic area. This type of illness was reported to be significantly less frequent in the rural population than in the urban population.

**Conclusion:**

The presence of both fever and dyspnea as both initial and main symptoms is a poor prognostic factor for COVID-19, regardless of age, sex, and demographic areas. In addition, women reported lower levels of fever and dyspnea, requiring minimal emergency assistance and fewer hospitalization, and a lower rate of mortality than men. During a COVID-19 infection follow-up, subjects in rural areas seem to have less access to medical care than those in urban areas.

## Introduction

The COVID-19 pandemic caused by the novel severe acute respiratory syndrome coronavirus 2 (SARS-CoV-2) was first identified in Wuhan, China, in December 2019 and spread rapidly around the world (1, 2). Currently, the pandemic has spread to more than 200 countries, areas, or territories, with ~464 million cases and ~6 million deaths worldwide (3). Spain is one of the most affected countries in Europe. Spain had a prevalence of 3.2 million cases since the pandemic was declared until March 2021, with 69,609 deaths. Especially, in Aragon, there were 106,860 confirmed cases and 3,265 deaths at the same time (4).

Since the start of the pandemic, the symptoms described have varied greatly, from asymptomatic to severe pneumonia and death. Symptoms are highly non-specific, with initial symptoms including fever, dry cough, asthenia, expectoration, dyspnea, sore throat, headache, myalgia or arthralgia, chills, nausea or vomiting, nasal congestion, diarrhea, hemoptysis, and conjunctival congestion (5–8). Subsequent studies added new symptoms such as neurological disorders and cardiological, otorhinolaryngological, dermatological, and hematological symptoms (9–11). In the clinical course of the first outbreak in China, of the laboratory-confirmed cases, 80% had mild or moderate symptoms, 13.8% had severe symptoms with dyspnea, tachypnea, and low oxygen saturation, and 6.1% had severe symptoms with respiratory failure, septic shock, and/or multiorgan failure (12).

On the other hand, different studies reported that both sex and age were risk factors for a poor prognosis of COVID-19 infection (13). Indeed, the mortality rate was 18.8% for patients aged over 80 years, whereas the overall mortality rate is estimated to be as high as 5% (14, 15). Another study reported that male sex, crackles, a higher fraction of inspired oxygen, and functionality were independent risk factors for mortality in elderly patients hospitalized for COVID-19 (16). In addition to sex and age, our research group identified demographic areas as another risk factor for the worst progression after COVID-19 infection in a retrospective study of 6,286 positive cases from Aragon (Spain), corresponding to the first wave of COVID-19 (17).

The response of the National Health Service (NHS), especially to primary healthcare (PHC), has been crucial in containing COVID-19. The first medical contact for COVID-19 cases is normally a PHC team, which determines the severity of symptoms, manages the follow-up of mild cases, and organizes hospital referrals for moderate-to-severe cases (17). Therefore, one of the key factors that can especially help the primary care physician during this pandemic is the prediction of the progression of a new coronavirus infection. Despite this, few studies analyzed the predictive nature of progression based on initial and predominant symptoms, and most of them are studies conducted in China (18). Several studies reported the impact that the COVID-19 pandemic has had on the PHC (19, 20). SESPAS 2022 (21) reports on the major and important changes that the PHC in Spain has undergone. A battery of measures, such as the lack of accessibility, the implementation of telephone consultation, telematic remote care, access to medical records, the promotion of electronic prescriptions, and the electronic issuance of sick leave and high work, were introduced. A study conducted in 16 European countries from primary care, reporting the effect of the pandemic on changes in patient consultations in European rural primary care, is worth mentioning. They found significant differences between countries in adopting measures for PHC. Remote teleconsultation is highly appreciated by health professionals and patients alike, but the most common form of remote consultation is still telephone consultation (22). Additionally, even fewer studies are based on strictly out-of-hospital symptom data. Therefore, the objective of this study was to analyze the prognosis [emergency assistance, hospitalization, intensive care unit (ICU) admission, and death] Fof patients with COVID-19 according to baseline and main symptoms, age, and sex in two different demographic regions according to the clinical data reported by a PHC.

## Materials and methods

### Study design

We conducted a retrospective study analyzing anthropometric, clinical, symptomatology, and demographic variables of all patients who tested positive for COVID-19, followed by general practitioners (GPs) from two health centers, one urban and the other in Aragon (Spain). The data were collected from 11 February 2021 to 2 March 2021 using the Electronic Clinical Record of Aragon's Health System and integrated into a fully anonymized database. The study protocol was approved by the Clinical Research Ethics Committee of Aragón (PI20/262).

### Subjects

In the present study, all subjects who over 18 years were registered in urban or rural areas of Aragon (Spain), diagnosed with COVID-19, and who were monitored by a primary care doctor in one of the health centers. Being diagnosed with COVID-19 was determined by a positive result in a polymerase chain reaction (PCR) test. Each patient included in the study was recruited in terms of sex, age at the time of infection, type of contact, baseline symptoms, primary and secondary symptoms, emergency assistance, hospitalization, ICU admission, and death. Type of contact refers to whether it is known through which contact the COVID-19 infection was contracted, categorizing it into the following options: family contact, social contact, work contact, social health field (which includes infection contracted at a health center or in a social and institutional center as a residence for the elderly), unknown, and not included or provided. Symptomatology was categorized into the following primary symptoms: (a) asymptomatic; (b) fatigue, which includes asthenia, weakness, apathy, loss of mobility, and malaise; (b) fever, which includes fever higher than 37.8°C, feverishness, diathermic sensation, and chills; (c) myalgia, which includes myalgia, arthralgia, low back pain, neck pain, and back pain; (d) cough, which includes cough, productive cough, dry cough, chest pain, pleuritic pain, and flank pain; (e) sore throat, which includes odynophagia, aphonia, dysphonia, plaques, congestion, mucus, rhinorrhea, sneezing, catarrh, pharyngeal process, and rhinitis; (f) headache; (g) anosmia, which includes anosmia, hyposmia, ageusia, and dysgeusia; (h) diarrhea; (i) dyspnea which includes dyspnea, respiratory discomfort, respiratory distress, and respiratory failure; (j) anorexia, which includes anorexia and hyperoxia; (k) irregular lung auscultation (ILA), which includes crackles, rhonchi, and wheezing; (l) somnolence, which includes drowsiness, lethargy, disorientation, confusion, and tremor at rest; (m) bacteremia, which includes bacteremia, sepsis, prerenal renal failure, and multi-organ failure; (n) hypotension, which includes hypotension and dizziness; and (o) skin disorders, which includes skin lesions, acrocyanosis, and aphthae.

### Statistical analysis

Continuous variables are expressed as mean ± standard deviation (SD) or median (25–75th percentile) as applicable, and categorical (nominal) variables are reported as percentages of the total sample. Differences between independent variables were calculated by the Student's *t*-test or the Mann–Whitney *U* test, as appropriate, while categorical variables were compared using the chi-squared test. Differences between independent variables were calculated by adjusting for sex and age using a linear regression model. Multivariable regression based on death, hospitalization, or emergency assistance was analyzed using binary logistic regression. All statistical analyses were performed with R version 3.5.0 (23), and their significance was set at a *p*-value < 0.05.

## Results

Of the 21,337 inhabitants aged over 18 years in both rural and urban health centers, 433 subjects (2.02%) were diagnosed with COVID-19 and followed up by a GP. Regarding clinical characteristics, we found fever, cough, and sore throat as the most prevalent baseline symptomatology. In addition, subjects reported 20.09% of emergency assistance, 14.332% of hospital admissions, 0.6% of ICU admissions, and 8.78% of deaths (Table 1).

**Table 1:** Clinical characteristics according to the demographic area.

		**Total (*N* = 433)**	**Rural areas (*N* = 219)**	**Urban areas (*N* = 214)**	***p*** **-value**
Age, years		57.5 ± 22.6	61.3 ± 23.8	53.8 ± 21.4	**< 0.001**
Men, *n* (%)		185 (42.73)	103 (47.0)	82 (38.3)	0.083
Baseline symptomatology	Asymptomatic, *n* (%)	92 (21.25)	54 (24.7)	38 (17.8)	0.069
	Fatigue, *n* (%)	68 (15.70)	20 (9.13)	48 (22.4)	**< 0.001**
	Fever, *n* (%)	137 (31.64)	66 (30.1)	71 (33.2)	0.425
	Myalgia, *n* (%)	41 (9.47)	8 (3.65)	33 (15.4)	**< 0.001**
	Cough, *n* (%)	69 (15.94)	24 (11.0)	45 (21.0)	**0.003**
	Sore throat, *n* (%)	69 (15.94)	14 (6.39)	55 (25.7)	**< 0.001**
	Headache, *n* (%)	55 (12.70)	14 (6.39)	41 (19.2)	**< 0.001**
	Anosmia, *n* (%)	36 (8.31)	9 (4.11)	27 (12.6)	**0.009**
	Diarrhea, *n* (%)	26 (6.0)	2 (0.91)	24 (11.2)	**< 0.001**
	Dyspnea, *n* (%)	27 (6.24)	8 (3.65)	19 (8.88)	**0.002**
	Anorexia, *n* (%)	4 (0.92)	0 (0)	4 (1.87)	0.995
	Irregular lung auscultation, *n* (%)	2 (0.46)	1 (0.45)	1 (0.47)	0.552
	Drowsiness, *n* (%)	7 (1,62)	3 (1.37)	4 (1.87)	0.276
	Bacteremia, *n* (%)	0 (0)	0 (0)	0 (0)	NA
	Hypotension, *n* (%)	1 (0.23)	1 (0.45)	0 (0)	0.998
	Skin changes, *n* (%)	1 (0.23)	0 (0)	1 (0.47)	0.998
Main symptomatology	Asymptomatic, *n* (%)	109 (25.17)	54 (24.7)	55 (23.4)	0.649
	Fatigue, *n* (%)	71 (16.40)	16 (7.31)	55 (25.7)	**< 0.001**
	Fever, *n* (%)	127 (29.33)	68 (31.1)	59 (27.6)	0.373
	Myalgia, *n* (%)	46 (10.62)	7 (3.19)	39 (18.2)	**< 0.001**
	Cough, *n* (%)	81 (18.71)	22 (10.0)	59 (27.6)	**< 0.001**
	Sore throat, *n* (%)	61 (14.09)	12 (5.47)	49 (22.9)	**< 0.001**
	Headache, *n* (%)	47 (10.85)	11 (5.02)	36 (16.8)	**0.001**
	Anosmia, *n* (%)	35 (8.08)	10 (4.56)	25 (11.7)	**0.042**
	Diarrhea, *n* (%)	32 (7.39)	1 (0.45)	31 (14.5)	**< 0.001**
	Dyspnea, *n* (%)	31 (7.16)	14 (6.39)	17 (7.94)	0.068
	Anorexia, *n* (%)	6 (1.39)	0 (0)	6 (2.80)	0.992
	Irregular lung auscultation, *n* (%)	0 (0)	0 (0)	0 (0)	NA
	Drowsiness, *n* (%)	4 (0.92)	2 (0.90)	3 (1.40)	0.450
	Bacteremia, *n* (%)	1 (0.23)	0 (0)	1 (0.47)	0.991
	Hypotension, *n* (%)	0 (0)	0 (0)	0 (0)	NA
	Skin changes, *n* (%)	2 (0.46)	0 (0)	2 (0.93)	0.995
Emergency assistance, *n* (%)		87 (20.09)	49 (22.4)	38 (17.8)	0.841
Hospitalization, *n* (%)		62 (14.32)	33 (15.1)	29 (13.6)	0.213
ICU admission, *n* (%)		3 (0.69)	3 (1.37)	0 (0)	0.997
Death, *n* (%)		38 (8.78)	18 (8.22)	20 (9.35)	**0.002**
Mode of transmission	Family, *n* (%)	144 (33.26)	47 (21.5)	97 (45.3)	
	Laboral, *n* (%)	33 (7.62)	17 (7.76)	16 (7.48)	
	Social, *n* (%)	31 (7.16)	12 (5.47)	19 (8.87)	**< 0.001**
	Social health field, *n* (%)	101 (23.33)	69 (31.5)	32 (15.0)	
	Not included or provided. *n* (%)	94 (21.71)	73 (33.3)	21 (9.81)	
	Unknown, *n* (%)	30 (6.93)	1 (0.4)	29 (13.6)	

Quantitative variables are expressed as means ± standard deviations (SD), except for variables not following normal distribution that are expressed as medians (interquartile ranges). Qualitative variables are expressed as percentages. The p-value was calculated by Student's t-test, Chi-square, or a binary regression model adjusted by age and sex, as appropriate.

NA, Not applied; ICU, Intensity care unit. Bold values indicate that the results are statistically significant.

Of the 4,880 inhabitants belonging to rural health centers, 219 (4.48%) were diagnosed with COVID-19 and followed up by a primary care doctor. Of the 16,457 inhabitants belonging to urban health centers, 214 (1.30%) were diagnosed with COVID-19 and followed up by a primary care doctor. Compared to subjects in urban areas, those diagnosed with COVID-19 in rural areas were significantly older in age and were predominantly men (Table 1). Additionally, we found that subjects in rural areas reported fewer baseline and main symptoms. Table 1 shows clinical characteristics and baseline symptomatology according to the demographic area. We found that patients in rural areas reported significantly lower levels of fatigue, myalgia, cough, sore throat, headache, anosmia, diarrhea, and dyspnea than those in urban areas (*p* < 0.001 in all cases, except the cases of cough, anosmia, and dyspnea symptoms, respectively). Concerning the primary symptoms, subjects in rural areas reported significantly lower levels of fatigue, myalgia, cough, sore throat, headache, anosmia, and diarrhea than those in urban areas (*p* < 0.001, except in headache and anosmia). Of the total number of patients in both rural and urban health centers, we found that 87 (19.6%) patients required emergency assistance, 62 (13.9%) were hospitalized, three required ICU admission, and 38 (8.57%) died. Despite the different reported symptoms, we did not find significant differences between the two groups in terms of emergency assistance, hospitalization, or ICU admission. Only the percentage of deaths was significantly higher in patients diagnosed in the urban population than in those diagnosed in the rural population (*p* = 0.002, Table 1).

Additionally, Table 1 shows the modes of transmission according to the demographic area. We found that patients in rural areas reported the social health field as the most frequent mode of transmission, while patients in urban areas reported family contact as the most common mode of transmission (*p* < 0.001). In addition, we observed that the modes of transmission were better recollected by those in urban areas, where they were not provided in only under 10% of the cases, compared to those in rural areas, where more than 30% of the cases were unaccounted for.

Of the 433 patients with COVID-19 in both rural and urban health centers, 185 (42.7%) were men, 248 (57.3%) were women, and subjects from both sexes had similar ages (*p* = 0.863, Table 2). Regarding baseline and main symptoms, women reported lower levels of fever and dyspnea as baseline symptoms and a higher percentage of anosmia as the main symptom than men (*p* = 0.025, *p* = 0.036, and *p* = 0.041, respectively, Table 2). Concerning the main symptoms, we only found differences in the prevalence of anosmia. This symptom was significantly higher in women than in men (*p* = 0.027, Table 2). However, despite a few differences with regard to symptomatology, men needed more emergency assistance, higher hospitalization rates, and higher mortality rates than women (*p* = 0.002, *p* = 0.001, and *p* = 0.009, respectively, Table 2).

**Table 2:** Clinical characteristics according to sex.

		**Men (*N* = 185)**	**Women (*N* = 248)**	***p*** **-value**
Age, years		57.8 ± 22.5	57.4 ± 23.2	0.863
Baseline symptomatology	Asymptomatic, *n* (%)	37 (20.0)	55 (22.2)	0.479
	Fatigue, *n* (%)	36 (19.5)	32 (12.9)	**0.025**
	Fever, *n* (%)	68 (36.7)	69 (27.8)	**0.041**
	Myalgia, *n* (%)	15 (8.11)	26 (10.5)	0.652
	Cough, *n* (%)	29 (15.7)	40 (16.1)	0.897
	Sore throat, *n* (%)	23 (12.4)	46 (18.5)	0.208
	Headache, *n* (%)	21 (11.4)	34 (13.7)	0.721
	Anosmia, *n* (%)	10 (5.41)	26 (10.5)	0.108
	Diarrhea, *n* (%)	12 (6.48)	14 (5.64)	0.426
	Dyspnea, *n* (%)	15 (8.11)	11 (4.44)	**0.036**
	Anorexia, *n* (%)	2 (1.08)	2 (0.80)	0.601
	Irregular lung auscultation, *n* (%)	0 (0.0)	2 (0.80)	0.995
	Drowsiness, *n* (%)	2 (0.54)	5 (2.02)	0.714
	Bacteremia, *n* (%)	0 (0.0)	0 (0.0)	NA
	Hypotension, *n* (%)	1 (0.54)	0 (0.0)	0.998
	Skin changes, *n* (%)	0 (0.0)	0 (0.0)	NA
Main symptomatology	Asymptomatic, *n* (%)	41 (22.2)	63 (25.4)	0.416
	Fatigue, *n* (%)	33 (17.8)	38 (15.3)	0.229
	Fever, *n* (%)	56 (30.3)	68 (27.4)	0.346
	Myalgia, *n* (%)	18 (9.73)	28 (11.3)	0.955
	Cough, *n* (%)	29 (15.7)	50 (20.2)	0.612
	Sore throat, *n* (%)	20 (10.8)	41 (16.5)	0.217
	Headache, *n* (%)	18 (9.73)	29 (11.7)	0.806
	Anosmia, *n* (%)	8 (4.32)	27 (10.9)	**0.027**
	Diarrhea, *n* (%)	13 (7.03)	19 (7.66)	0.806
	Dyspnea, *n* (%)	15 (8.11)	15 (6.05)	0.195
	Anorexia, *n* (%)	3 (1.62)	3 (1.21)	0.517
	Irregular lung auscultation, *n* (%)	0 (0.0)	0 (0.0)	NA
	Drowsiness, *n* (%)	1 (0.54)	4 (1.61)	0.360
	Bacteremia, *n* (%)	0 (0.0)	1 (0.40)	0.997
	Hypotension, *n* (%)	0 (0.0)	0 (0.0)	NA
	Skin changes, *n* (%)	0 (0.0)	1 (0.40)	0.997
Emergency assistance, *n* (%)		49 (26.5)	38 (15.3)	**0.002**
Hospitalization, *n* (%)		37 (0.20)	25 (10.1)	**0.001**
ICU admission, *n* (%)		3 (1.62)	0 (0.0)	0.997
Death, *n* (%)		21 (11.4)	17 (6.85)	**0.009**

Quantitative variables are expressed as means ± SD, except for variables not following normal distribution that are expressed as medians (interquartile ranges). Qualitative variables are expressed as percentages. The p-value was calculated by Student's t-test, Chi-square, or a binary regression model adjusted by age and demographic area, as appropriate.

NA, Not applied; ICU, Intensity care unit. Bold values indicate that the results are statistically significant.

Table 3 shows multivariate regression based on hospitalization, death, or emergency assistance as a function of baseline symptomatology. In all cases, the absence of baseline fever, being young, living in rural areas, or being a woman are protective factors with respect to the probability of being admitted to the hospital, dying, or requiring emergency care. In addition, in the case of hospitalization, the absence of dyspnea is a protective factor against being admitted to the hospital. The case of mortality is especially striking, where we noticed that the absence of fever and dyspnea, a younger age, living in rural areas, and being a woman explained more than 48% of the chances of death due to COVID-19 (Table 3).

**Table 3:** Multivariate regression based on hospitalization, deaths, or emergency assistance as a function of baseline symptomatology.

	**OR**	**95% CI**	***p*** **-value**	***R^2^*** **Nagelkerke**
**Hospital** **admission**				
No fever	0.402	0.215–0.750	0.004	0.283
No dyspnea	0.276	0.111–0.687	0.006	
Age, years	1.051	1.032–1.069	< 0.001	
Rural demographic area	0.822	0.437–1.548	0.545	
Sex, women	0.438	0.237–0.811	0.008	
**Death**				
No fever	0.436	0.184–1.031	0.058	0.482
No dyspnea	0.595	0.180–1.965	0.395	
Age, years	1.136	1.091–1.187	< 0.001	
Rural demographic area	0.280	0.114–0.684	0.009	
Sex, women	0.367	0.156–0.685	0.016	
**Emergency** **assistance**				
No fever	0.490	0.290–0.829	0.008	0.194
Age, years	1.039	1.026–1.052	< 0.001	
Rural demographic area	0.989	0.589–1.661	0.922	
Sex, women	0.497	0.298–0.829	0.008	

Multivariable binary logistic regression with the hospitalization, deaths, or emergency assistance as dependent variables.

OR, Odd ratio; CI, Confidence interval of OR.

In the same regard, the main symptoms revealed an increased likelihood of ending up hospitalized or requiring urgent care (Table 4). Patients who do not have fever, are younger, live in rural areas, and are women have a lower probability of requiring hospitalization, with these factors accounting for up to 25% of the probability. Similarly, the need for emergency assistance depends on the presence of fever, dyspnea, age, demographic area, and sex. We found that the absence of fever and dyspnea, younger age, living in rural areas, and female sex are protective factors requiring emergency assistance, with these factors accounting for a probability of up to 24% (Table 4).

**Table 4:** Multivariate regression based on hospitalization or emergency assistance as a function of main symptomatology.

	**OR**	**95% CI**	***p*** **-value**	***R^2^*** **Nagelkerke**
**Hospital admission**				
No fever	0.474	0.257–0.874	0.017	0.252
Age, years	1.054	1.037–1.071	< 0.001	
Rural demographic area	0.681	0.370–1.252	0.217	
Sex, female	0.391	0.214–0.714	0.002	
**Emergency** **assistance**				
No fever	0.354	0.207–0.608	< 0.001	0.243
No dyspnea	0.274	0.120–0.626	0.002	
Age, years	1.035	1.022–1.049	< 0.001	
Rural demographic area	0.996	0.583–1.1699	0.379	
Sex, women	0.486	0.268–0.820	0.007	

Multivariable binary logistic regression with hospitalization, deaths, or emergency assistance as dependent variables.

OR, Odd ratio; CI, Confidence interval of OR.

## Discussion

Our study analyzed exclusively external hospital data, collected from two health centers, one in a rural area and another in an urban area, showing that the presence of fever and dyspnea, as both initial and main symptoms, in addition to age and male sex, are risk factors for a worse prognosis after COVID-19 infection. In addition, it is interesting to highlight that the Spanish public system is practically universal; therefore, the results can be extrapolated to the general population. We found huge differences between the symptoms reported according to the demographic area and sex, with greater symptoms reported by the male and urban populations.

According to clinical symptoms, laboratory indicators, and imaging findings, COVID-19 is classified as mild, normal, severe, and critical. A subject is determined to have severe COVID-19 if any of the following diagnosis criteria are satisfied: shortness of breath with a respiratory rate >30 times/min, oxygen/saturation >93% at rest, or arterial blood oxygen partial pressure < 300 mmHg (24). Pulmonary imaging showed that the lesions significantly progressed within 24–48 h, and >50% were managed according to severity (25). According to the analysis of existing clinical characteristics, patients with severe COVID-19 tend to have dyspnea after 1 week, and in some cases, moderate to low fever. Severe cases were more likely to rapidly deteriorate, affected by septic shocks, metabolic acidosis, and coagulopathy, generating a multiorgan failure (26). Therefore, it is necessary to predict the prognosis of COVID-19 as early as possible. For this reason, numerous articles attempted to ascertain the initial clinical symptoms that allowed the identification of high-risk patients. For example, the meta-analysis by He et al. shows that fever and dyspnea, among other symptoms, frequently occurred in patients with severe COVID-19 pneumonia, concluding that these patients should be closely monitored to prevent disease deterioration (18). Similarly, the study conducted by Richardson et al. on 5,700 hospitalized patients between March and April 2020 in 12 hospitals in the New York City Area reported that more than 30% of patients had fever and more than 15% presented dyspnea at triage (26). In our case, we found that the presence of fever and dyspnea, in both initial and main symptoms, are significantly associated with hospitalization, emergency assistance, and death, regardless of age, sex, and demographic area. In other words, fever and dyspnea are independent risk factors to worsen the prognosis for COVID-19. Therefore, patients who exhibit these symptoms should be closely monitored to prevent disease deterioration, particularly in the elderly and in male patients. With the pandemic now achieving the status of a flu, these symptoms could be incorporated into the detection protocols for severe COVID-19 so that they would be screening questions to categorize and to be able to identify the patient who are at the greatest risk.

In addition, we separately analyzed initial and main symptomatology with the initial hypothesis that the evolution of symptoms could vary substantially in the same person. However, we found that those patients who have fever and dyspnea as baseline symptoms also have them as their main symptoms and are those that require more medical attention and have a worse prognosis of COVID-19. Although, fever and dyspnea have already been reported by other studies as alarm symptoms, which indicated a worse evolution after COVID-19 infection (18, 26, 27). Our study shows for the first time that the presence of these symptoms among the initial symptoms is also an indicator of a worse prognosis and evolution due to COVID-19.

Both age and sex have been considered as independent risk factors for a poor prognosis of COVID-19 in several studies, such as the study conducted by Grasselli et al. (28) on 3,988 patients with COVID-19 hospitalized in ICUs in Italy. In this study, the authors reported that both age and male sex are independent risk factors associated with mortality. In this regard, the OpenSAFELY cohort study, with 17 million adult patients in England, demonstrated that COVID-19-related deaths were associated with male sex, regardless of age, low income, smoking, pre-existing diseases, and ethnicities (29). Sex susceptibility has been analyzed in different pathologies, showing that women seem to be the strongest sex, facing diseases such as hypertriglyceridemia (HTG), type 2 diabetes, cardiovascular disease (CVD), or chronic obstructive pulmonary disease, among others, which has usually been attributed to a better immune system and hormonal system. However, although women indeed present a lower prevalence of CVD, a later age for the diagnosis of diseases such as diabetes or HTG, and lower mortality, they also suffer from a situation of underdiagnosis in most diseases (30–33). Some articles hypothesized that it would be role of androgens to detect the progression and prognosis after COVID-19 infection, since the spike proteins of the SARS-COV2 virus would use the transmembrane protease serine 2 for entry into the host (34). Nonetheless, most studies found differences in the immune system of each sex (35, 36). In fact, one study carried out by Takahashi et al. published in the journal *Nature* demonstrated that male patients had higher plasma levels of innate immune cytokines, such as IL-8 and IL-18, along with more robust induction of non-classical monocytes. In contrast, female patients had more robust T-cell activation than male patients during the SARS-CoV-2 infection. Furthermore, they found that a poor T-cell response was negatively correlated with the patients' age and was associated with a worse disease outcome in male patients but not in female patients (37). With respect to age, several studies reported that age is probably the most important prognostic risk factor for COVID-19, and this effect could be explained by a greater number of comorbidities associated with elderly people, in addition to a more weakened immune system (35, 38–43). In our study, we found similar results to those previously published, reporting that age and male sex are independent risk factors for a worse prognosis after COVID-19 infection to show that the elderly and male sex have significantly higher probabilities of requiring hospitalization, emergency assistance, or even death.

An interesting point is the high percentage of registered deaths, which exceeds 8% in both rural and urban health centers. This percentage is much higher than the current mortality rate, which slightly exceeds 1% in Aragon and is below 1% across Spain nationally (44, 45). These differences can be explained because the data correspond to the period prior to the full vaccination of the Spanish population, which has largely decreased mortality after infection with COVID-19 (46, 47). Indeed, the mortality rates in the first wave exceeded 30% in Spain and Aragon (17), which is similar to other European countries such as Italy, France, or the UK (48, 49).

Regarding demographic differences, we found that patients in rural areas reported much fewer symptoms than those in urban areas. These differences could be explained by a combination of factors, including epidemiological and population factors, such as population density, distribution by age and sex, the prevalence of underlying diseases, and the lower access and health resources of the rural population (50). However, we found that the percentage of deaths was significantly higher in urban areas than in rural areas, which was contradictory to what was expected, since the rural population also has a high proportion of men and subjects who are old. These percentages could be explained by the different number of inhabitants belonging to each health center. If we determine the percentage of mortality according to all inhabitants belonging to each health center, almost 5,000 inhabitants in rural areas vs. more than 16,000 inhabitants in urban areas, we found that the percentage of deaths is significantly higher in rural areas than in urban areas, which is in line with the results that were previously published (17). Regarding the studies and reports conducted in PHC, the impact of mortality in Spain was one of the highest in the world. According to data from the updates of the Ministry of Health of Spain, mortality in the first wave of the pandemic was 20% in people over 70 years of age. In the first wave of the pandemic, mortality was 21.5% in a PHC cohort conducted on people aged over 65 years. In another study conducted in the same period, age was observed to be the main factor associated with mortality in patients with COVID-19, along with male sex, diabetes, dyslipidemia, and heart failure (21, 51).

Finally, another interesting point to comment on is the different modes of transmission between urban and rural regions. Patients in rural areas were found to be infected mostly in health centers, followed by the family environment. However, for patients in urban areas, the majority route of contagion was through family. It is true that the distance to hospitals is greater in rural areas than in urban areas, and the logistic accessibility is more difficult; however, these data obtained in our study may be more related to the sociodemographic characteristics of the rural population. In Spain, 28.5% of the rural population is over 65 years of age, while in urban areas it is only 18.5%. The aging of this population entails a greater use of social health centers by the elderly and, with it, more possibilities of infection in these centers. Magallón-Botaya et al. described in their study that rural areas are usually further away from urban cities and are inhabited by an elderly population with greater social isolation and less need to travel for work, so it is very likely that the elderly population has been especially cautious in the social and family environment in the face of the pandemic (17, 45). Similarly, it should be noted that there is a higher percentage of subjects in rural areas who have not identified the form of contagion, which would be another point of confirmation of low access to the available health system in rural areas.

### Limitations

Our study has some limitations. First, one of the limitations lies in the data collection, that is, data were obtained through the Electronic Medical Record of Aragon's Health System, which relies on the data collected by the primary care physician. In addition, it is important to mention the training received by professionals in both rural and urban centers as well as the similar number of professionals in each of them, so there seems to be no bias of this type in the registry. Therefore, this same point allows us to compare the registry systems of two health centers with different demographic regions and confirm the previous results. Second, the data collection only includes individuals who have been followed up by the GP, thus excluding cases that went directly to the emergency room or individuals coming from nursing homes, which normally have their own clinic. Therefore, the mortality percentages may not correctly represent those of the population, although this was not one of the objectives of the present study. Despite this, this study provides important information on the COVID-19 pandemic in our environment, analyzing the demographic factor and pointing out those individuals that are at a greater risk of complications and symptoms that deserve more exhaustive control to avoid a bad evolution.

## Conclusions

In conclusion, the presence of fever and dyspnea as both initial and main symptomatology is a worse prognostic factor for COVID-19, regardless of age, sex, and demographic areas. Fewer women reported fever and dyspnea at baseline symptomatology, as well as lower emergency and hospital assistance and mortality, than men, which indicates that the immune system of women is more efficient in managing a COVID-19 infection. On the other hand, subjects in rural areas reported less initial and main symptomatology than those in urban areas, as well as lower determination of its form of contagion, indicating that this population has received less follow-up. This could partly explain the higher mortality experienced in both rural and suburban areas among the population of Aragon, Spain.

## Data availability statement

The raw data supporting the conclusions of this article will be made available by the authors, without undue reservation.

## Ethics statement

The studies involving human participants were reviewed and approved by Clinical Research Ethics Committee of Aragón (PI20/262). Written informed consent for participation was not required for this study in accordance with the national legislation and the institutional requirements.

## Author contributions

BO-B, RM-B, and JG-A drew up the research design. BO-B, DL-I, and SF-M developed the study and coordinated the fieldwork. IL-M did the quantitative analysis. FM-L and RM-B have helped with project coordination. BO-B and CB-M wrote the manuscript. BO-B is the principal investigator of the project. All authors reviewed the manuscript content and approved the final version for submission.
